# RNA-binding proteins as targets for pain therapeutics

**DOI:** 10.1016/j.ynpai.2018.01.003

**Published:** 2018-01-31

**Authors:** June Bryan de la Peña, Zachary T. Campbell

**Affiliations:** Department of Biological Sciences, University of Texas Dallas, Richardson, TX 75080, United States

**Keywords:** RNA-binding proteins, Pain

## Abstract

RNA-protein interactions permeate biology. Transcription, translation, processing, and mRNA decay all hinge on widespread use of regulatory information decoded by RNA-binding proteins. The final committed step of protein synthesis, translation, is intimately linked to nociceptor excitability. Understanding the factors that control translation is essential as nociceptor plasticity is a hallmark of persistent pain. Here, we review the growing body of evidence for widespread involvement of RNA-binding proteins in pain. Many of the relevant factors have been implicated in post-transcriptional and translational mechanisms of mRNA control. We propose that recent advances in the development of RNA-based therapeutics provide a potential means to exploit our current understanding of liaisons between RNAs and proteins for therapeutic purposes.

## Introduction

Transcription results in the production of ribonucleic acid (RNA) and RNA can be translated into protein. This framework provides a simplistic view into how instructions present in the genomic blueprint manifest in complex and dynamic patterns of protein expression. The final committed step in this process, translation, is intimately linked to nociceptor plasticity and the development of pain hypersensitivity (Alvarez et al., 2000; Alvarez, 2001; Price and Geranton, 2009; Khoutorsky et al., 2015). Understanding the factors that influence translation is important as nociceptor plasticity both requires protein synthesis and is a hallmark of persistent pain (Ferrari et al., 2013a).

As the essential intermediate in protein synthesis, messenger RNA (mRNA) is subject to regulation at virtually every level. Precise control of mRNA processing, stability, translation, and localization dictates the efficiency, timing, and site of protein production. For instance, mRNA stability spans nearly four orders of magnitude ranging from minutes to months (Goldstrohm and Wickens, 2008). Similarly, translation can occur in spatially defined regions of a cell such as the cell body or in axons (Brittis et al., 2002; Bramham and Wells, 2007). Integral to local translation is polarity in the cytoplasmic distribution of mRNAs (Medioni et al., 2012). This information is encoded by cis-acting regulatory elements with variable strength, number, and positioning. The interaction between these elements and their protein partners gives rise to mRNA function and is ultimately the mechanism that trans-acting factors utilize to decode regulatory information.

**Fig. 1.**
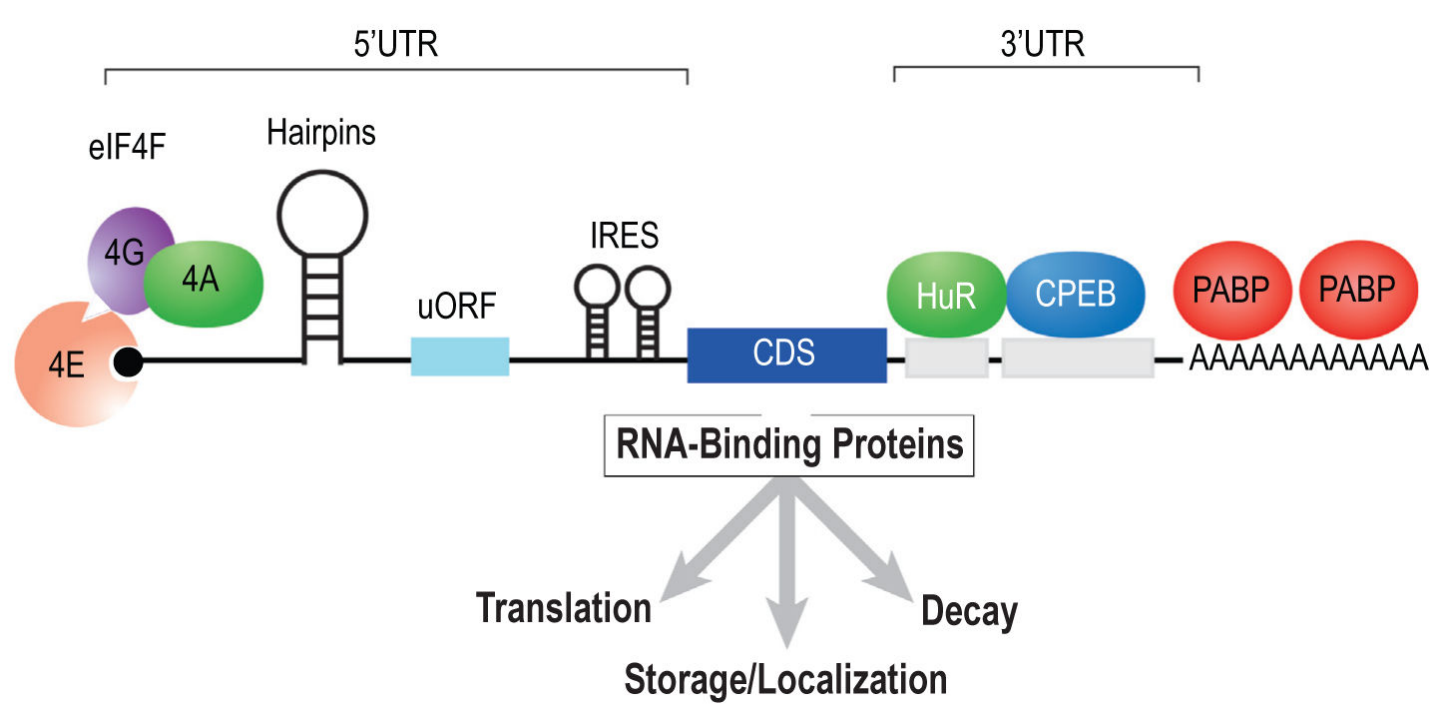
RNA-protein interactions on a model mRNA. The coding sequence (CDS) is flanked by a 5′ and 3′ untranslated region (UTR). The UTR contains sequences and structures that are bound by proteins such as HuR and CPEB. Additionally, hairpins, IRES, and uORFs in the 5′ UTR can modulate protein expression. The m7G cap and Poly(A) tail are bound by the eIF4F complex and PABP respectively.

Regulatory sequences and structural elements occur throughout a transcript and serve a crucial role in specification of mRNA function (see Fig. 1). For instance, translation initiation is generally orchestrated through interactions that require proteins bound to the 5′ 7-methylguanylate (m7G) cap of an mRNA (Yanagiya et al., 2012). The cap is bound by a multi-protein complex containing eukaryotic translation initiation factors (eIF)4A, eIF4G, and eIF4E (collectively referred to as eIF4F) (Gingras et al., 1999). eIF4E is the subunit responsible for cap binding and is subject to dynamic regulation through sequestration with protein partners and phosphorylation (Sonenberg et al., 1978; Matsuo et al., 1997). Adjacent to the m7G cap is the 5′ untranslated region (UTR). The UTR serves as a repository for regulatory information including secondary structural elements, internal ribosomal entry sequences (IRES), upstream open reading frames (uORFs) and RNA-binding protein motifs. IRES elements bypass the requirement for specific translation factors and thus mediate cap-independent translational control (Hellen and Sarnow, 2001). 5′ UTRs can also contain hairpin structures and pseudoknots which make translation less efficient from the main open reading frame. These secondary structures can be unwound by helicases such as eIF4A (Feoktistova et al., 2013; Garcia-Garcia et al., 2015). The 5′ UTR can also contain uORFs which generally reduce translation of the downstream reading frame. uORFs may encode bioactive peptides as has been recently demonstrated in immunity (Starck et al., 2016). Like the 5′ UTR, the 3′ UTR is also resplendent with motifs for regulatory protein complexes. These factors can enhance or reduce protein synthesis, but majority of these mechanisms appear to be repressive - implying that the default status of translation favors protein production. A dynamic feature of mRNA metabolism is modulation of poly-adenosine(A) tail length (Goldstrohm and Wickens, 2008). After transcription, the mRNA possesses a long poly(A) tail. This tail can undergo regulated shortening through a process called deadenylation. RNA-binding proteins influence poly(A) tail length through recruitment of cytoplasmic poly(A) polymerases or deadenylases. MicroRNAs (miRNA) can also elicit deadenylation (Valencia-Sanchez et al., 2006). Loss of the poly(A) tail can stimulate decapping and decay (Chen and Shyu, 2011).

In this review, we summarize recent progress into conserved mechanisms of mRNA control implicated in pain. We focus first on the trans-acting factors involved in RNA recognition. We describe new potential players based on analysis of cis-acting elements found in key transcripts. Finally, we discuss the potential use of RNA-based inhibitors for understanding underlying mechanisms of chronic pain and as a new source of pain therapeutics.

## RNA-binding proteins that are involved in pain

Several RNA-binding proteins have been identified to play a role in pain through various animal models of chronic pain conditions (see Table 1). The functions of these RNA-binding proteins range from mRNA cap recognition, stabilization, stimulation, repression, and even decay.

### Cap-binding protein

The 5′ m7G cap of an mRNA is bound by the cap-binding protein eIF4E. eIF4E is controlled by protein-partners and phosphorylation. In dorsal root ganglion (DRG) neurons, the pro-inflammatory mediators nerve growth factor (NGF) and interleukin-6 (IL-6) promote translation through convergent effects on eIF4F association with the m7G cap (Melemedjian et al., 2010). In both cases, nascent protein synthesis is enhanced presumably due to more efficient translation initiation. The kinase, mechanistic/mammalian target of rapamycin (mTOR), promotes cap-dependent translation in part through negative regulation of eIF4E-binding proteins (Beretta et al., 1996). mTOR binds to Raptor and other protein partners to form the rapamycin-sensitive mTOR complex 1 (mTORC1), which phosphorylates the eIF4E-binding protein 1 (4E-BP1) allowing the release of eIF4E and formation of the eIF4F complex. Accordingly, one consequence of mTOR inhibition is reduced binding of the eIF4F complex to the m7G cap (Mathews et al., 2007). At least three additional lines of evidence suggest that eIF4E activity is relevant to pain. First, systemic dosing of the mTORC1 inhibitor, temsirolimus or Torinl, reduces mechanical and cold hypersensitivity induced by nerve injury in mice (Obara et al., 2011). Second, deletion of 4E-BP1 in mice increases mechanical hypersensitivity (Khoutorsky et al., 2015). Third and finally, eIF4E phosphorylation promotes the development of nociceptor sensitization (Moy et al., 2017), although the precise function of this phosphorylation in enhanced sensitization is not entirely known. Collectively, these experiments illuminate the mechanistic role of eIF4E and the mRNA cap in both acute and persistent pain and has been reviewed in detail elsewhere (Khoutorsky and Price, 2017).

### ARE-binding proteins

Adenylate-uridylate-rich elements (AU-rich elements; AREs) are abundant in the 3′ UTR of mRNAs encoding cytokines and immuneresponsive genes (Chen and Shyu, 1995). The presence of an ARE has a major impact on mRNA stability (Shaw and Kamen, 1986). A variety of proteins facilitate ARE function through direct interactions with mRNA including members of the Hu family (a.k.a. ELAV-like RNA-binding proteins). Mechanistically, Hu proteins can either enhance RNA stability or increase recruitment of the mRNA to the polysome (Antic et al., 1999). This is a unique feature of Hu proteins as opposed to other ARE-binding proteins (Bolognani and Perrone-Bizzozero, 2008). The ubiquitously expressed member of the family, HuR, has numerous roles related to cellular stress response, while the neuronal members of the family, HuB, HuC, and HuD, serve vital functions in plasticity and brain development (Hinman and Lou, 2008). Members of the Hu family appear to facilitate pain in mice. Antisense oligonucleotide (ASO) depletion of HuD via intrathecal injection reverts persistent pain in an animal model of antiretroviral therapy (Sanna et al., 2015). Similarly, intrathecal ASO depletion of HuR attenuates mechanical allodynia in a model of autoimmune encephalomyelitis (Sanna et al., 2017). These studies suggest that ARE binding proteins and the 3′ UTR promote nociceptive signals likely at the level of RNA stability.

### Cytoplasmic polyadenylation element binding (CPEB) protein

CPEBs are a well-established paradigm in activity-dependent translational regulation (Richter, 2007). CPEBs recognize AU-rich sequences in the 3′ UTR and can either repress or stimulate polyadenylation depending on its phosphorylation status (Hodgman et al., 2001). CPEB knockdown by intrathecal ASO injection inhibits plasticity in rat hyperalgesic priming models (Bogen et al., 2012). Similarly, CPEB depletion reduces mechanical allodynia in an animal model of HIV-related neuropathic pain (Iida et al., 2016). Furthermore, mice with deletion of the CPEB3 gene are hypersensitive to noxious heat (Fong et al., 2016). These experiments all point to a role of CPEB in nociceptive plasticity. In addition, local activation of the CPEB target CaMKIIα induces hyperalgesic priming and mechanical hyperalgesia (Ferrari et al., 2013b; Wu et al., 2014). This result suggests a key role for local translation of CaMKIIα in the transition from acute to chronic pain. Given that CPEBs are broad-spectrum pleiotropic regulators with hundreds to thousands of targets, there are undoubtedly additional targets of CPEB in axons (Fernandez-Miranda and Mendez, 2012). Their identification will expedite efforts to fully appreciate the full repertoire of potential targets of CPEB and their potential relationship to pain.

### Fragile X mental retardation protein (FMRP)

The underlying cause of Fragile X syndrome is loss of function of the RNA-binding protein, Fragile X mental retardation protein (FMRP). FMRP regulates synaptic plasticity and binds to a subset of mRNA and directly to the L5 protein on the 80S ribosome (Ashley et al., 1993; Chen et al., 2014). Fmr1-knockout (Fmr1-KO) mice show decreased responses to ongoing pain and a lag in the onset of injury-induced allodynia (Price et al., 2007). mTOR inhibition of formalin and DHPG induced nociception was also impaired in Fmr1-KO mice suggestive of core deficits in translational controls that mediate plasticity (Price et al., 2007). In addition, IL-6-induced peripheral sensitization is strongly blunted in Fmr1-KO mice suggestive of an underlying defect in local protein synthesis (Asiedu et al., 2011). Given that FMRP is directly bound to ribosomes and that loss of FMRP promotes translation elongation (Darnell et al., 2011), an unanswered question remains as to how FMRP affects axonal translation in afferent fibers and if this contributes to abnormal plasticity.

### Poly(A) binding proteins (PABPs)

In addition to general roles in promoting translation and mRNA stability, PABPs participate in additional mRNA control mechanisms including mRNA storage during cellular stress, mRNA export, mRNA quality control via nonsense-mediated decay, and miRNA-mediated translational repression (Gorgoni and Gray, 2004). These functions hinge on the association of PABPs with Poly(A) tails. A growing body of evidence suggests the Poly(A) tail is essential in pain. For instance, the small molecule cordycepin that disrupts polyadenylation inhibits pain plasticity after local delivery in hyperalgesic priming models (Ferrari et al., 2013a). Similarly, the end result of CPEB activation is enhanced cytoplasmic poly(A) addition. In recent work, we have shown that RNA-based decoys of the Poly(A) tail, which bind PABP with high affinity *in vitro*, block mechanical hyperalgesia and priming induced by pro-inflammatory cytokines, capsaicin, or incision injury (Barragán-Iglesias et al., 2018). PABPs are found in axons and the Poly(A) decoy, when delivered locally, blocks priming. This contributes additional evidence to the notion that disruption of local protein synthesis can impair hyperalgesic priming and suggests that PABPs are required for axonal translation (Melemedjian et al., 2010; Wong et al., 2010; Ferrari et al., 2013a).

### Other RBPs with potential roles in pain

Additional RBPs are linked, either directly or indirectly, with various pain states, particularly those that contribute in local protein synthesis in sensory neurons (see Table 2). Sensory neurons are vital mediators of nociceptive sensitization. Local protein synthesis in nociceptor terminals or their distal axons has been implicated in promoting hyperexcitability and producing pain sensitization (Obara et al., 2012). Inhibition of activity-dependent translation in peripheral axons blocks the development of persistent plasticity as measured by the presence of hyperalgesic priming. This suggests that onset of chronic pain requires regulated local protein synthesis. Thus, understanding basic mechanisms that drive pain sensitization is crucial for the identification of potential targets for chronic pain treatment. Several RNA-binding factors implicated in local translation may play important roles in pain. For instance, the double stranded dsRNA-binding protein, Staufen, is expressed in peripheral sensory neurons and may play a role in axonal mRNA trafficking (Price et al., 2006). The zinc finger protein 36 like 2 (ZFP36L2) RBP positively regulates axonal integrity in mature DRG neurons by destabilization of the RE1 Silencing Transcription Factor (REST) (Cargnin et al., 2014). Heterogeneous nuclear ribonucleoprotein (hnRNPs) can also be found in axons (hnRNP R) and serve as post-transcriptional regulators of opioid receptor expression (hnRNP H1 and F) (Song et al., 2012; Dombert et al., 2014). Targeting these axonally localized RBPs might be beneficial for peripheral neuropathic pain.

Genome-wide approaches suggest that a remarkable amount remains to be uncovered about mechanisms of non-coding RNA, local translation, RNA-decay, and their relationship to pain. For example, noxious stimuli alter patterns of alternative splicing as well as changes in 3′ end utilization (Donaldson and Beazley-Long, 2016; Hirai et al., 2017). The use of a distal 3′ end isoform of Nav 1.8 mRNA imparts axonal localization after chronic injury. While it is not clear what proteins regulate this differential processing event or axonal localization, cis-acting elements found in the alternate isoform hint at potential roles for Pumillio homolog 2 (Pum2) and polypyrimidine tract-binding protein 1 (PTBP1) (Hirai et al., 2017). The underlying mechanisms and functional significance of this phenomenon remain to be elucidated.

Injury appears to alter non-coding RNA function. For instance, pain impacts miRNA expression patterns and genetic defects in miRNA processing impact pain thresholds (Zhao et al., 2010; Sakai and Suzuki, 2014; Jiangpan et al., 2016). Along these lines, RBPs that play a role in miRNA biogenesis such as Argonaut (Ago), TAR RNA-binding protein, and Dicer might also contribute to pain (McDonald and Ajit, 2015). Long non-coding RNAs and the m6a modification have also been recently implicated in pain further adding to the narrative that diverse RNA-based mechanisms contribute to nociception (Zhao et al., 2013; Weng et al., 2018).

Of the thousands of changes in RNA structure and abundance that are correlated with pain, the challenge becomes establishing causation. Because proteins both regulate and collaborate with RNAs to mediate their function, new approaches that disrupt this interface have a tremendous potential to illuminate our understanding of pain mechanisms *in vivo.*

## New therapeutic tools

While the specificity of many RNA-binding proteins is known, rational design of competitive inhibitors is challenging given the unstable nature of RNAs (Sachs et al., 1987; Ray et al., 2013). Tremendous progress on transcription factors has been made based on DNA mimics of transcription factor consensus binding elements (Morishita et al., 1998; Mann and Dzau, 2000; Kang et al., 2008; Melemedjian et al., 2014). RNA is rapidly degraded by exonucleases *in vivo* but can be stabilized through chemical modifications to the 2′ hydroxyl and the phosphodiester backbone (Dias and Stein, 2002; Kole et al., 2012). We recently demonstrated that such modifications can be introduced into short 12 base RNA oligos. These chemical modifications have minimal effects on equilibrium dissociation constants and increase half-life from hours to over 10 days in serum-containing media (Barragán-Iglesias et al., 2018). As a proof of concept for the approach, the compounds, termed specificity derived competitive inhibitor oligonucleotides or SPOT-ONs, were added to axons *in vitro.* The RNAs are taken up and localize throughout axons and the cell body. A SPOT-ON directed against the Poly(A) binding protein with selectivity *in vitro* attenuates translation initiation and functions as a robust anti-hyperalgesic *in vivo.* Because this approach targets the RNA-binding region of a given target, there is no inherent reliance on processing factors from the host. An additional advantage of the SPOT-ON approach is the ability to inhibit entire protein families that bind similar, if not identical sequences, to enact similar functions. By way of comparison, genetic dissection of paralogues gene families is extremely laborious and may result in negative results due to functional compensation. The simplicity of design suggests that this method will be broadly applicable for mechanistic studies of RNA-binding proteins *in vivo.* SPOT-ONs complement existing methods for protein reduction that are suitable for *in vivo* studies including siRNAs and ASOs. These experiments will be critical to elucidate new mechanisms of action with the coveted goal of improved therapies for chronic pain sufferers.

## Figures and Tables

**Table 1:** Regulation of RBPs in different models of chronic pain conditions.

RBP	Biological functions	Depletion strategy	Pain model	Outcome	Citation
eukaryotic translation initiation factor 4E (eIF4E)	Recognizes and binds the 5′ cap of an mRNA. It is an essential component of the eIF4F complex that promotes translation.	Knock-in (KI) mutation of the eIF4E phosphorylation site (serine 209) in mice (eIF4E^S209A^ mice).	Mechanical and thermal hyperalgesia induced by pronociceptive (NGF, IL6) and inflammatory (carrageenan) factors, and of injury (SNI) in eIF4E^S209A^ mice. Assessment of increases in neuronal excitability induced by NGF and IL6.	Pronociceptive and inflammatory factors produce decreased mechanical and thermal hypersensitivity, decreased affective pain behaviors, and strongly reduced hyperalgesic priming in eIF4E^S209A^ mice. Cold hypersensitivity following peripheral nerve injury and NGF- and IL6-induced increases in neuronal excitability were also attenuated in eIF4E^S209A^ mice.	Moy et al. (2017)
HuD or ELAV Like RNA Binding Protein 4 (Elavl4)	A member of the Hu family that plays a role in neuronal development and plasticity.	Intrathecal antisense oligonucleotide (ASO). Single injection every 24 h for a total of 3 injections.	2′,3′-dideoxycytidine (ddC)-evoked painful neuropathy in mice, a model of antiretroviral neuropathy.	HuD silencing reverted ddC-induced pain hypersensitivity.	Sanna et al. (2015)
HuR or ELAV Like RNA Binding Protein 1 (Elavl1)	Ubiquitously expressed member of the Hu family. Positively regulates the stability of many target mRNAs, including several cytokines, and is involved in the maintenance of inflammation and in the proper functioning of the immune system.	Intrathecal ASO. Single injection every 4 days, for a total of 4 injections.	Mouse model (female only) of relapsing-remitting experimental autoimmune encephalomyelitis (RR-EAE), an experimental model of Multiple Sclerosis.	ASO treatment completely attenuated hind paw mechanical allodynia and thermal hyperalgesia developed by RR-EAE mice.	Sanna et al. (2017)
Cytoplasmic polyadenylation element binding protein (CPEB)	Promotes the elongation of the poly(A) tail of mRNA by recruiting all the molecular components necessary to catalyze polyadenylation.	Intrathecal ASO. A dose of 40 μg/day for seven consecutive days	Rats treated with carrageenan and subsequently challenged by prostaglandin E2 (PGE2). Rats treated with the selective PKCε agonist, ψεRACK, and subsequently challenged by PGE2	ASO-treated rats did not show enhanced and prolonged hyperalgesic response to PGE2. ASO-treated rats did not show hyperalgesic response to PGE2. Priming by ψεRACK, can be prevented but not reversed by CPEB ASO	Bogen et al. (2012)
		Intrathecal ASO. A dose of 40 μg/day and was administered for 3 consecutive days	Neuropathic pain induced by HIV envelope protein gp120 combined with antiretroviral drug (ddC).	ASO-treated rats displayed reduced mechanical allodynia.	Iida et al. (2016)
		Cpeb3 gene knockout (Cpeb3-KO)	Assessment of sensory (thermal and mechanical) and hyperalgesic [Complete Freund’s adjuvant (CFA)-induced inflammatory pain model] responses in Cpeb3-KO mice.	Cpeb3-KO mice demonstrated hypersensitivity to noxious heat. In the CFA-induced inflammatory pain model, Cpeb3-KO mice showed normal thermal hyperalgesia and transiently enhanced mechanical hyperalgesia.	Fong et al. (2016)
Fragile X mental retardation protein (FMRP)	Binds to mRNAs and is involved in transporting them to distal sites in cells while repressing their translation.	Fmr1 gene knock-out (Fmr1-KO)	Mechanical and thermal threshold assessment, formalin-induced nociception, and nerve injury-induced allodynia in Fmr1-KO mice. *In vitro* spinal cord preparation to examine the incidence of wind-up responses (plasticity) in ascending sensory fibers after repetitive C-fiber stimulation.	Fmr1-KO mice showed decreased responses to ongoing nociception, a delay in the development of peripheral nerve injury-induced allodynia, and a near absence of wind-up responses.	Price et al. (2007)
Poly(A) binding protein (PABP)	PABP binds the poly(A) tail. It protects RNA from deadenylation and stimulates translation initiation by bridging the poly(A) tail to the eIF4F complex.	PABP inhibition by a chemically modified RNA-based competitive inhibitor (SPOT-ON).	Mechanical hyperalgesia and priming induced by pro-inflammatory cytokines (NGF or IL6), capsaicin, or incision in mice.	Local delivery of the PABP SPOT-ON blocked mechanical hyperalgesia induced by either pro-inflammatory cytokines, capsaicin, or incision.	Barragán-Iglesias et al. (2018)

**Table 2:** Other RBPs that might have a role in pain.

RBP	Relevant findings	Citation
Staufen (Stau)	Peripheral sensory neurons express the RNA binding and transport protein, Staufen, and this protein localizes to DRG axons. Stau plays a role in trafficking RNA to sensory axons and, therefore, are attractive targets for therapeutic intervention in sensory dysfunctions, including pain.	Price et al. (2006)
Zinc finger protein 36 like 2 (ZFP36L2)	ZFP36L2 promotes axonal integrity in mature DRG neurons by destabilizing the RE1 Silencing Transcription Factor (REST).	Cargnin et al. (2014)
Heterogeneous nuclear ribonucleoproteins (hnRNPs)	hnRNP R is localized in axons and axon terminals of embryonic and postnatal mouse motoneurons. hnRNP H1 and F function as post-transcriptional repressors of the *mu*-opioid receptor.	Dombert et al. (2014)
Polypyrimidine tract-binding protein 1 (PTBP1)	PTBP1 and PUM2 binding sequences were found in the extended 3′ UTR sequence of Nav1.8 mRNA present exclusively in the injured axon.	Song et al. (2012)
Pumillio homolog 2 (PUM2)		
Argonaut (Ago) TAR RNA-binding protein Dicer	These RBPs play a role in miRNA biogenesis. miRNAs are promising players in pain management.	McDonald and Ajit (2015)
